# Comprehensive Analysis of Subtype-Specific Molecular Characteristics of Colon Cancer: Specific Genes, Driver Genes, Signaling Pathways, and Immunotherapy Responses

**DOI:** 10.3389/fcell.2021.758776

**Published:** 2021-11-29

**Authors:** Fangjie Hu, Jianyi Wang, Minghui Zhang, Shuoshuo Wang, Lingyu Zhao, Hao Yang, Jinrong Wu, Binbin Cui

**Affiliations:** ^1^Department of Colorectal Surgery, Harbin Medical University Cancer Hospital, Harbin Medical University, Harbin, China; ^2^Department of Pathology, Harbin Medical University, Harbin, China; ^3^Department of Oncology, Chifeng City Hospital, Chifeng, China; ^4^Department of Radiation Oncology, Inner Mongolia Cancer Hospital & Affiliated People’s Hospital of Inner Mongolia Medical University, Hohhot, China; ^5^Department of Anaesthesiology, The First Affiliated Hospital of Harbin Medical University, Harbin, China

**Keywords:** colon cancer, molecular subtypes, driver gene, immune infiltration, immune checkpoint

## Abstract

Colon cancer is a complex, heterogeneous disease. The Colorectal Cancer Subtyping Consortium reported a novel classification system for colon cancer in 2015 to better understand its heterogeneity. This molecular classification system divided colon cancer into four distinct consensus molecular subtypes (CMS 1, 2, 3, and 4). However, the characteristics of different colon cancer molecular subtypes have not been fully elucidated. This study comprehensively analyzed the molecular characteristics of varying colon cancer subtypes using multiple databases and algorithms, including The Cancer Genome Atlas (TCGA) database, DriverDBv3 database, CIBERSORT, and MCP-counter algorithms. We analyzed the alterations in the subtype-specific genes of different colon cancer subtypes, such as the RNA levels and DNA alterations, and showed that specific subtype-specific genes significantly affected prognosis. We also explored the changes in colon cancer driver genes and representative genes of 10 signaling pathways in different subtypes. We identified genes that were altered in specific subtypes. We further detected the infiltration of 22 immune cell types in four colon cancer subtypes and the infiltration level of primary immune cells among these subtypes. Additionally, we explored changes in immune checkpoint genes (ICGs) and immunotherapy responses among different colon cancer subtypes. This study may provide clues for the molecular mechanism of tumorigenesis and progression in colon cancer. It also offers potential biomarkers and targets for the clinical diagnosis and treatment of different colon cancer subtypes.

## Introduction

Colon cancer is the third most common cancer and second leading cause of cancer-related death globally (Sung et al., 2021). Clinically, colon cancer is primarily classified according to its histopathological features, including tumor size, grade, and disease stage. However, this classification method ignores the heterogeneity of colon cancer. The molecular characteristics of colon cancer tissues of the same pathological type may show significant differences, preventing traditional pathological classifications from accurately distinguishing the biological aspects of colon cancer. From 2012 to 2014, six teams reported representative molecular subtype systems (Schlicker et al., 2012; Budinska et al., 2013; De Sousa et al., 2013; Marisa et al., 2013; Sadanandam et al., 2013). However, because of differences in patient cohorts, sequencing platforms, bioinformatics analysis methods, and data analysis in different studies, each subtype system has different interpretations of the molecular classification of colon cancer. In 2015, based on six subtyping systems, the Colorectal Cancer Subtyping Consortium identified four consensus molecular subtypes (CMS1, CMS2, CMS3, and CMS4), providing the most robust classification system for colon cancer to date (Guinney et al., 2015). The implementation of molecular classification in the clinical decision-making of colon cancer is crucial to solve various clinical problems in colon cancer progression. Presently, different treatment strategies can be formulated according to different molecular classifications in the clinic, including applying microsatellite instability (MSI) and BRAF mutations to predict clinical treatment and prognosis (Van Cutsem et al., 2016; Benson et al., 2018, 2020).

The present study explored the molecular characteristics of four colon cancer subtypes, such as the RNA level, DNA level, and immune infiltration level. We also explored the differences in driver genes and representative genes of 10 signaling pathways in the different colon cancer subtypes and assessed their influence on patient prognosis. Additionally, we evaluated the immune therapy response of four subtypes, including the immune checkpoint gene expression level and immunophenoscore. We aimed to explore the potential differences between different subtypes and identify potential biological therapeutic targets to provide new ideas for the clinical treatment of colon cancer.

## Materials and Methods

### Data Source

We downloaded colon cancer data (41 normal samples and 471 colon cancer samples) from the UCSC Xena database^1^ across the following four genomic platforms: RNA expression, gene mutations, copy number variation, and single nucleotide polymorphisms (SNPs).

### Classifier Analysis of Colon Cancer

The CMSclassifier developed by Bionetworks was applied to classify the four molecular subtypes of colon cancer samples—CMS1, CMS2, CMS3, and CMS4 (Guinney et al., 2015). The DESeq R package was used to analyze the differential expression of mRNAs, lncRNAs, and miRNAs between different colon cancer subtypes, and a *p*-value < 0.01 was considered statistically significant (Anders and Huber, 2010). Differentially expressed RNAs were identified in cancer samples and normal samples. Next, each subtype-specific RNA was identified by calculating the differential expression of each gene between a certain subtype and other subtypes and considering the intersection of the differentially expressed genes between every two groups as the gene specifically expressed by the subtype. For example, the analysis of the differences was performed between CMS1 and CMS2, between CMS1 and CMS3, and between CMS1 and CMS4. Next, the intersection of genes with significant differences was finally defined as specific RNA of CMS1. Regarding the cutoff parameters of the differentially expressed genes, a *p*-value < 0.01 and log_2_FC > 1.5 or log_2_FC < −1.5 were defined as significantly different genes. The screening methods for specifically expressed lncRNAs and miRNAs were consistent with those described above. Hierarchical clustering analysis was conducted using the R package pheatmap. Each cluster was calculated using the average expression level of subtype-specific RNAs in different subtypes. Metascape^2^ was used to perform gene ontology (GO) analysis.

### Prognostic Analysis of the Four Colon Cancer Subtypes

Kaplan-Meier survival curves were used to explore the potential link between subtype-specific genes and OS. The median was used as the cutoff for high or low expression of subtype-specific RNAs.

### Analysis of Driver Genes in Each Subtype

Fifteen recognized driver gene prediction algorithms were used to identify the driver genes using the DriverDBv3 database^3^ (Liu et al., 2020). The driver gene represents a gene detected by more than five algorithms. The expression levels of the driver genes were subjected to variance analysis, and we analyzed the alterations in driver genes in different subtypes.

### DNA Alterations Among the Four Subtypes

We analyzed the DNA alterations (copy number variation, gene mutations, and SNPs) of different genes in four colon cancer subtypes, such as subtype-specific RNA, driver genes, and representative genes from 10 oncogenic pathways.

### Tumor Mutation Burden of Different Colon Cancer Subtypes

The tumor mutation burden (TMB) is the total number of mutations per million bases in tumor tissue, such as somatic gene coding errors, base substitutions, insertions, and deletions (Lawlor et al., 2021). The TMB data of colon cancer were downloaded from the TCGA database. The colon cancer samples of each subtype were divided into low- and high-TMB groups based on the median values. We compared the difference in survival between the low- and high-TMB groups of each subtype using Kaplan–Meier analysis, and the *p-*value was calculated using the log-rank test.

### Representative Gene Analysis in Classic Pan-Cancer Pathways

The classic pan-cancer atlas was downloaded from PathwayMapper^4^ and included 10 pathways: (1) cell cycle, (2) Hippo, (3) Myc, (4) Notch, (5) NRF2, (6) PI3K, (7) RTK/RAS, (8) TGF-β, (9) TP53, and (10) Wnt signaling (Bahceci et al., 2017). All mutation data of colon cancer samples were obtained from the cBioPortal database^5^. We evaluated the gene mutations and copy number variations (CNVs) of all essential representative genes in the 10 pathways. The sum of gene mutation and copy number variation was identified as the altered frequency of representative genes in the pathway.

### Analysis of Immune Cell and Fibroblast Infiltration Levels

CIBERSORT and MCP-counter algorithms were used to predict the levels of infiltrating immune cells and fibroblasts in colon cancer samples (Newman et al., 2015; Becht et al., 2016). Pearson correlation analysis was used to assess the correlation between immune cells and fibroblasts.

### Immune Therapy Score of Different Colon Cancer Subtypes

We used the immunophenoscore (IPS) from The Cancer Immunome Atlas to assess the immunotherapy response among the four subtypes of colon cancer (Charoentong et al., 2017). The immunophenoscore score primarily comprises four parts: MHC molecules (MHCs), effector cells (ECs), immune checkpoints (CPs), and immunosuppressive cells (SCs). Based on the expression of representative genes or gene sets of immune imaging, the IPS was calculated on a scale of 0--10. A higher IPS indicates more immunogenic tumors and a well-predicted response to anti-PD-1/PD-L1 therapy. The R code used is available at GitHub.^6^

### Immune Checkpoint Gene Analysis in Colon Cancer

Differential expression analysis of the immune checkpoint genes (ICGs) in each subtype of colon cancer was performed using the DESeq R package. A *p*-value < 0.01 was considered statistically significant. The samples of each subtype were divided into low and high expression groups according to the median values. Kaplan–Meier analysis was used to compare the difference in survival between the low and high expression groups of each subtype, and the *p-*value was calculated using the log-rank test.

## Results

### Identification of Molecular Subtype-Specific RNAs in Colon Cancer

**FIGURE 1:**
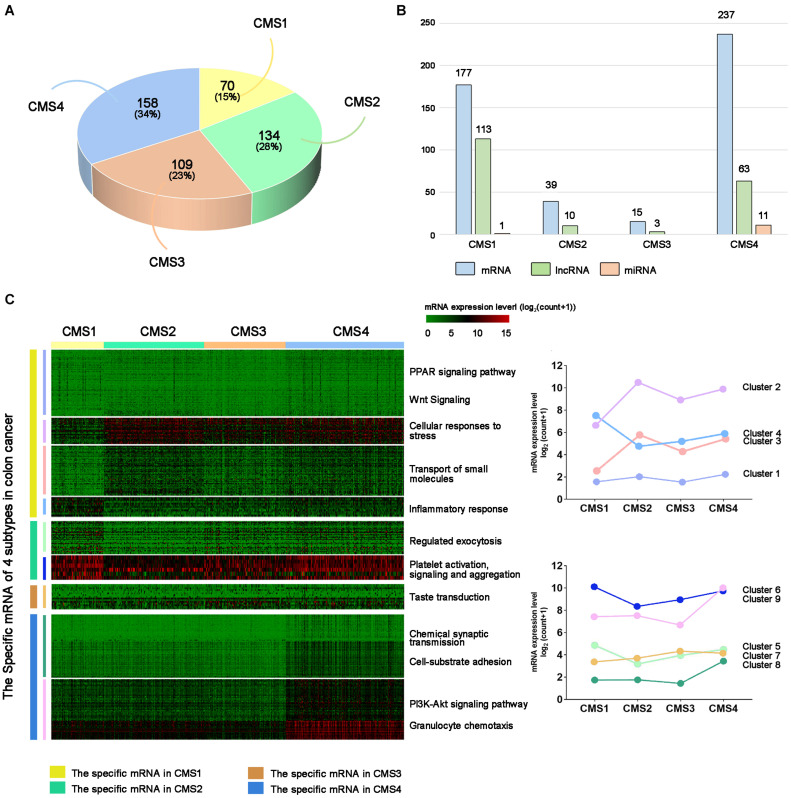
Subtype-specific RNA analysis of colon cancer. **(A)** Statistical analysis of colon cancer samples from each subtype. **(B)** Number of subtype-specific RNAs identified in each subtype. **(C)** The left panel shows hierarchical clustering analysis and GO analysis of subtype-specific RNAs in each subtype. Red indicates a high expression level, and green indicates a low expression level. The right panel shows the average values of subtype-specific RNAs in different clusters of the four subtypes.

First, we predicted the molecular subtypes of 471 colon cancer samples using the CMSclassifier method. The number of samples of each colon cancer subtype was as follows: CMS1: 70; CMS2: 134; CMS3: 109; CMS4: 158 (Figure 1A). We separately counted the number of RNAs specifically expressed in different subtypes (Figure 1B and Supplementary Table 1). Next, we explored the changes in the expression of specific mRNAs among different subtypes by performing hierarchical clustering analysis. Specific mRNAs identified in each subtype were divided into clusters with different biological characteristics (Figure 1C). The right panel shows the average value of each cluster, showing a significant difference among the four subtypes. We also analyzed the expression levels of lncRNAs and miRNAs in different subtypes (Supplementary Figures 1, 2). Based on these results, the expression level of RNAs specific to different subtypes was significantly different in a single subtype compared with other subtypes.

### Gene Mutations Among the Colon Cancer Subtypes

**FIGURE 2:**
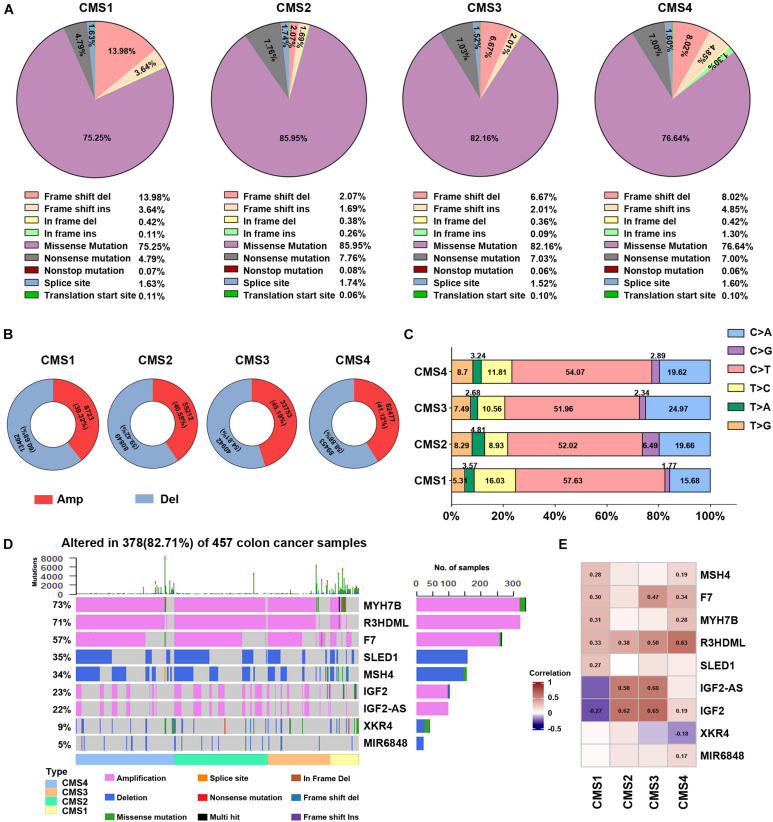
Analysis of DNA alterations in the four colon cancer subtypes. **(A)** Different classified categories of DNA mutations in each colon cancer subtype. **(B)** Number of copy number variations (CNVs) in each subtype. Red represents amplification, and blue indicates deletion. **(C)** Change in the SNV transversion ratios in the different subtypes. **(D)** Waterfall chart of the mutations in different subtype-specific genes. **(E)** Correlation analysis of subtype-specific RNAs and their modifications in the different subtypes. The numbers in the figure are correlation coefficients, where a negative value represents a negative correlation and a positive value represents a positive correlation.

To further explore the specific changes in different molecular subtypes in colon cancer, we evaluated the gene mutation status among the four subtypes of colon cancer, such as DNA mutations, CNVs, and SNPs. Missense mutations accounted for the highest proportion of all mutation types (Figure 2A), with the CMS2 subtype demonstrating the highest probability of missense mutations (85.95%) among the four subtypes. In the CMS1 subtype, the frameshift deletion mutation rate was the highest among the four types, at 13.98%, while the nonsense mutation rate was the lowest at 4.79%. Figure 2B shows the CNVs among the four different types. The CNV level of the four subtypes showed the lowest amplification in the CMS1 subtype and the highest deletions in the CMS4 subtype. Next, we compared the changes in SNPs among the different subtypes. The C > T transversion ratio among the four subtypes was the highest, exceeding 50%, reaching 57.63% in the CMS1 type (Figure 2C). The C > A and T > C conversion ratios were also higher in the four subtypes. Among them, the C > A conversion ratio was higher in the CMS3 type, and T > C was higher in CMS1. The CMS4 subtype showed more T > G transversions than the other subtypes. The proportion of CMS2 in the T > A and C > G subtypes was higher than that in the other subtypes. These results suggest specific differences in the gene mutation levels among the different subtypes. Next, we performed DNA mutation analysis on the subtype-specific genes obtained in Result 1. Nine genes were identified with a higher proportion of amplification and deletion—MYH7B, R3HDML, F7, SLED1, MSH4, IGF2, IGF2-AS, XKR4, and miR-6848 (Figure 2D). Further analysis of the correlation between the copy number variation of these genes and their RNA expression levels showed that most genes are related to their copy number variation. Among the four subtypes, the expression level of R3HDML was significantly positively correlated with its DNA amplification (Figures 2D,E).

### Subtype-Specific Genes Predicts the Prognosis of Patients With Colon Cancer

**FIGURE 3:**
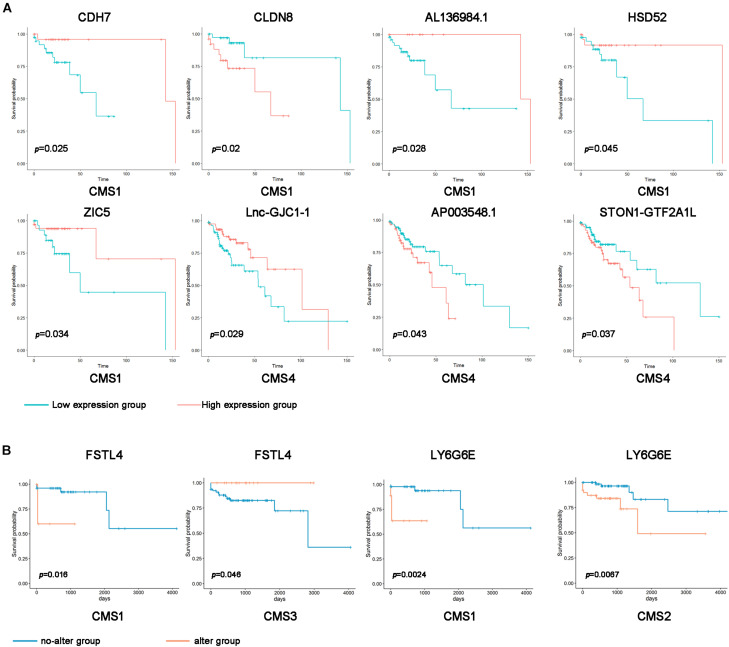
Correlation of subtype-specific RNA expression with overall survival in colon cancer patients. **(A)** Kaplan–Meier survival curves were generated for subtype-specific RNAs by comparing groups with high (red line) and low (blue line) gene expression. *p* < 0.05 according to the log-rank test. **(B)** Kaplan–Meier survival curve of the subtype-specific genes with gene mutations. Orange indicates the gene alteration group, and blue indicates the no-alteration group.

The above results suggest that the expression levels and mutations of specific RNAs in different subtypes show significant differences. Therefore, we speculate that changes in these genes may predict the prognosis of patients with different subtypes of colon cancer. Using the Kaplan–Meier method, we compared the overall survival of patients between the subtype-specific RNA high expression and low expression groups. The expression levels of many subtype-specific RNAs predicted the prognosis of patients with a single subtype. For example, in CMS1, the survival period of patients with high levels of CLDN8 was significantly shorter than that of patients with low levels of CLDN8. In CMS4, patients had lower levels of AP003548.1, and their survival time was longer (Figure 3A and Supplementary Figure 3; *p* < 0.05).

Additionally, we divided the samples of different types into a gene alteration group and a no-alteration group according to whether a mutation was present in the subtype-specific gene. Furthermore, we analyzed whether subtype-specific genetic changes affect the prognosis of patients. In the CMS1 sample, the survival rate of the abnormally changed FSTL4 and LY6G6E groups was significantly shorter than that of the entire group. By contrast, the unusually altered FSTL4 group in the CMS3 sample had longer survival rates than the no-alteration group (Figure 3B). These results indicate that the expression levels and mutations of these subtype-specific genes can be used to predict the prognosis of patients with different types.

### Identification of Driver Genes in the Colon Cancer Subtypes

**FIGURE 4:**
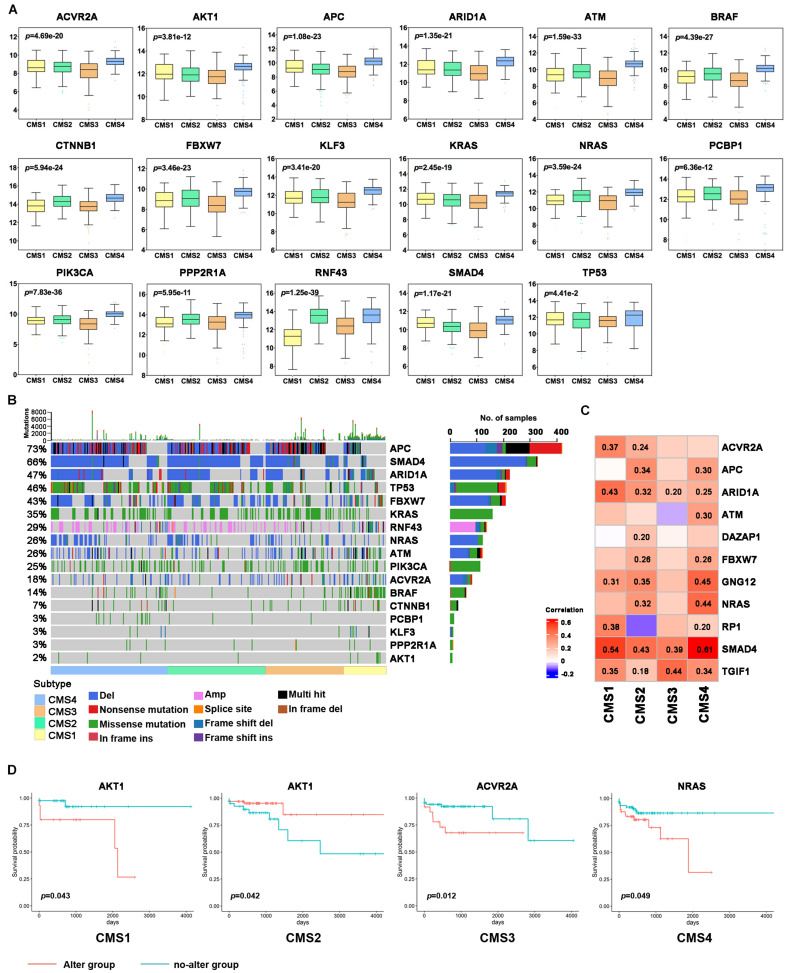
Analysis of driver genes in the different colon cancer subtypes. **(A)** Expression levels of the driver genes in each subtype. **(B)** Waterfall chart of the frequency of mutations in the driver genes in each subtype. **(C)** Correlation analysis of subtype-specific RNAs and their modifications in the different subtypes. **(D)** Kaplan–Meier survival curve of the driver genes with gene mutations. Red indicates the gene alteration group, and blue indicates the no-alteration group.

Tumor driver genes play critical roles in the occurrence and development of tumors. To further investigate the molecular features of different subtypes, we predicted 30 driver genes in colon cancer samples using the DriverDBv3 online database to explore the molecular characteristics of different subtypes (Supplementary Table 2). Differential analysis of the expression levels of driver genes in different subtypes revealed that the expression levels of almost all driver genes were significantly higher in CMS4 samples than in other subtype samples (Figure 4A and Supplementary Figure 4A). The expression of most driver genes was lower in CMS3. AXIN2, NEB, NRAS, RP1, RNF43, and other driver genes were significantly lower in CMS1 than in the other subtypes (Figure 4A and Supplementary Figure 4A). These results indicate that different driver genes may ultimately play different roles in the different subtypes. Regarding the different subtypes, driver genes may become decisive biomarkers and potential therapeutic targets.

**FIGURE 5:**
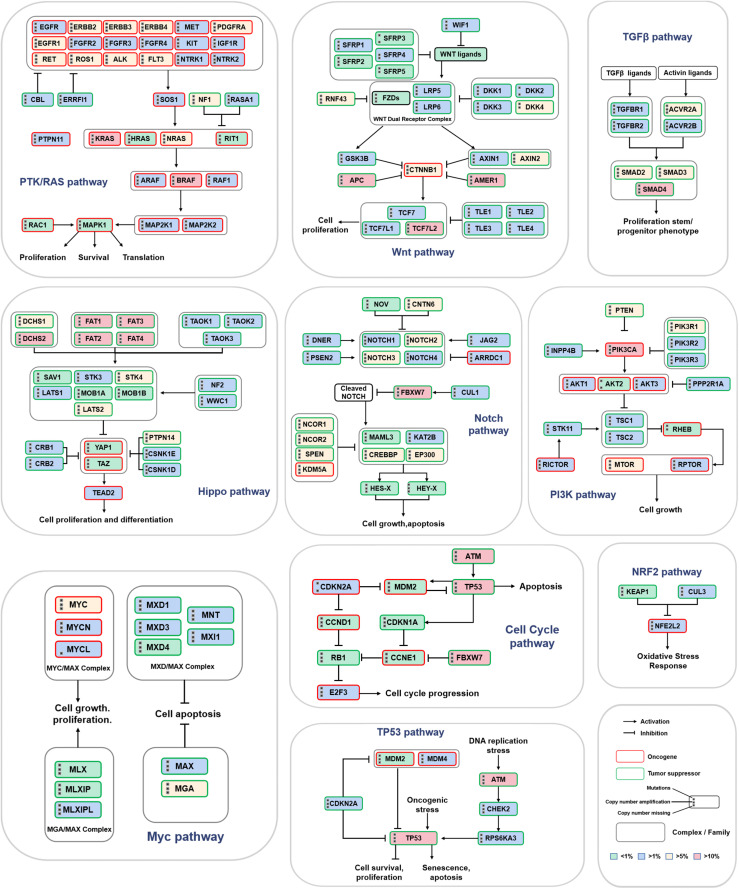
Mutation frequency of key genes in 10 classic signaling pathways. The three black dots on the left side of the gene name represent gene mutations, copy number amplifications and deletions. The red border represents oncogenes, and the green border indicates tumor suppressor genes. The intensity of the color represents the mutation frequency.

Next, we further analyzed the mutations of driver genes in the different subtypes. Most colon cancer samples showed abnormal changes in the APC, SMAD4, ARID1A, TP53, FBXW7, KRAS, RNF43, NRAS, ATM, and PIK3CA genes (Figure 4B). The BRAF gene is mainly expressed as missense mutations in CMS1 subtypes, and its mutation rate is significantly higher than that of the other subtypes. Except for CMS1, the mutation frequency of APC in the three other subtypes was more than 50%, and the mutation frequency of APC was the highest in CMS2. Missense mutations of KRAS and PIK3CA are more widespread in the CMS3 subtype, and the TP53 mutation is the most common in the CMS4 subtype. CMS4 subtype patients also had a higher proportion of SMAD4, ARID1A, and NRAS deletions. Next, we analyzed the correlation between the expression level of a driver gene and its mutation. The expression levels of many driver genes were positively correlated with their gene deletions, such as TGIF1, SMAD4, and ARID1A (Figure 4C and Supplementary Figures 4A,B). Additionally, to further study the potential cancer drivers in colon cancer progression, we evaluated 10 classical signaling pathways with frequently altered genes and essential cancer relative genes explored from these pathways derived from TCGA data. It was showed that critical oncogenes and tumor suppressor genes had higher mutation rates, such as KRAS, BRAF, APC, AMER1, TCF7L2, PIK3CA, FBXW7, DCHS2, FAT1, TP53, SMAD4, and ATM (Figure 5).

To further understand the significance of driving gene changes on the prognosis of different subtypes, we evaluated the alter of driver genes on the prognosis of patients with colon cancer. It was shown that the patients with CMS1 presenting alter of AKT1 had a shorter OS, and AKT1 no-alter of CMS2 had a prolonged OS (Figure 4D; *p* < 0.05). The survival rate of the abnormally changed KLF3 group in the CMS1 subtype was significantly shorter than that of the no-alteration group. The survival rate of the abnormally altered PCBP1 group in the CMS3 subtype was markedly shorter than that of the no-alteration group (Supplementary Figure 4C; *p* < 0.05).

### Differences in Immune Cell Infiltration Among the Different Subtypes

**FIGURE 6:**
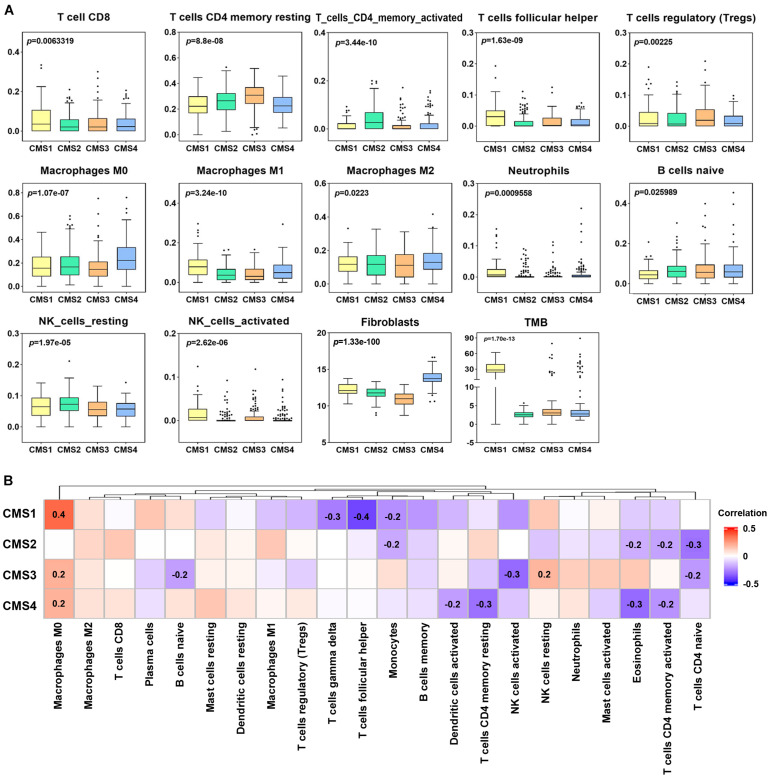
Levels of immune cell infiltration in different colon cancer subtypes. **(A)** Differential analysis of the immune cell infiltration level, fibroblasts and tumor mutation burden (TMB) in different colon cancer subtypes (*p* < 0.05). **(B)** Correlation analysis between fibroblasts and the levels of twenty-two infiltrating immune cell types in the different colon cancer subtypes. Red indicates a positive correlation, and blue indicates a negative correlation. The numerical value represents the degree of correlation (*p* < 0.05).

Immune cells are considered to be an important component of tumor microenvironment (TME), which plays an important role in tumor genesis, outcome, and treatment (especially immunotherapy) (Zhang et al., 2021). In addition to the analysis of subtype-specific genes alteration, an in-depth study of the clinical significance of immune cell infiltration levels in different subtypes is helpful to determine a clinical diagnosis and patient treatment. Therefore, we investigated the different invasion levels of 22 immune cell types in the 4 colon cancer subtypes. The infiltration level of these immune cell types was significantly different in the four subtypes of colon cancer (Figure 6A). CMS1 is an MSI immune subtype with the characteristics of immune infiltration and activation. The infiltration levels of CD8 T cells, T follicular helper cells, M1 macrophages, neutrophils, and NK activated cells in the CMS1 subtype were significantly higher than those in the other three subtypes. In the CMS2 subtype, the levels of infiltrating CD4 memory-activated cells and NK resting cells were higher than those in the other three subtypes. The CD4 memory resting cells were the highest in the CMS3 subtypes. The levels of infiltrating M0 macrophages and M2 macrophages were more elevated in the CMS4 subtype than in the other subtypes. Next, we examined the difference in the levels of infiltrating fibroblasts in the four subtypes, among which the levels of infiltrating fibroblasts in CMS4 were higher than those in the other subtypes. Thus, we further assessed the correlation between 22 immune cell types and fibroblasts in the different subtypes. Many T cells were negatively correlated with fibroblasts in the different subtypes, such as T follicular helper cells, which were negatively correlated with fibroblasts in CMS1 (Figure 6B). CD4 activated and resting T cells showed a negative correlation with fibroblasts in CMS4. Naïve CD4 cells were also negatively correlated with fibroblasts in CMS2 and CMS3.

Furthermore, we compared the TMB levels among the four subtypes and analyzed the correlation between the TMB and immune cell infiltration level. The level of TMB in the CMS1 subtype was significantly higher than that in the other subtypes (Figure 6A). Correlation analysis showed a significant correlation between the TMB and infiltration levels of specific immune cell types in the different subtypes. For example, the infiltration level of B memory cells was significantly positively correlated with the TMB in CMS1; T follicular helper cells and mast activated cells showed a positive correlation with the TMB in CM2. In CMS3, M1, and M2 macrophages showed a positive correlation with the TMB (Supplementary Figure 5).

**FIGURE 7:**
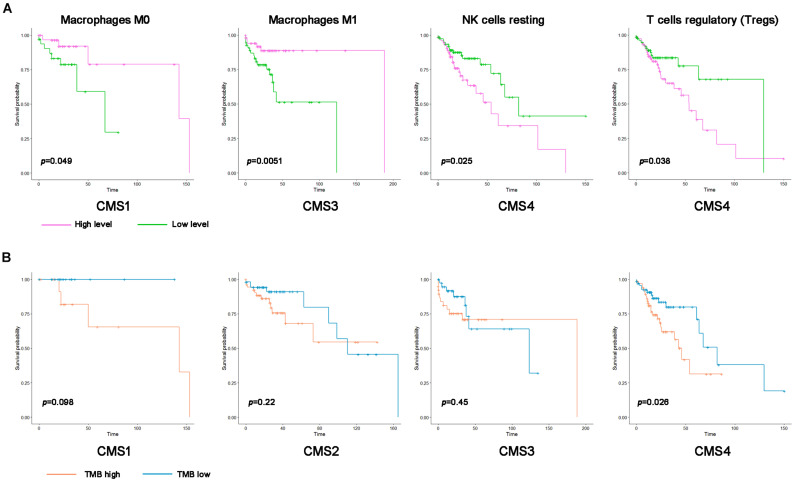
Kaplan–Meier survival curve of immune cells and the tumor mutation burden (TMB) in the different colon cancer subtypes. **(A)** Kaplan–Meier survival curve of the immune cell infiltration level in the different colon cancer subtypes (*p* < 0.05). Pink represents a high infiltration level, and green represents a low infiltration level. **(B)** Kaplan–Meier survival curve of the TMB in the different colon cancer subtypes. Blue represents the high TMB group, and orange represents the low TMB group.

Because the level of immune cell infiltration in different subtypes is significantly different, we speculate that its infiltration may be related to the prognosis of patients with different subtypes. Therefore, according to the level of immune cell infiltration, samples of various types were divided into high infiltration level and low infiltration level groups. Kaplan–Meier analysis was used to compare the prognostic differences between the two groups (Figure 7A). A low degree of resting NK cell and Treg cell infiltration indicated a better prognosis for patients with the CMS4 subtype. The group with high M0 macrophage infiltration and the CMS1 subtype showed a more extended survival period; high M1 macrophage infiltration in the CMS3 subtype group correlated with a better prognosis. We also analyzed whether the TMB level in the different subtypes affects the prognosis of patients. Only patients with CMS4 significantly differed in OS between the high TMB and low TMB groups (Figure 7B.).

### Immunophenogram Analysis Predicts the Response of the Different Molecular Subtypes to Immunotherapy

**FIGURE 8:**
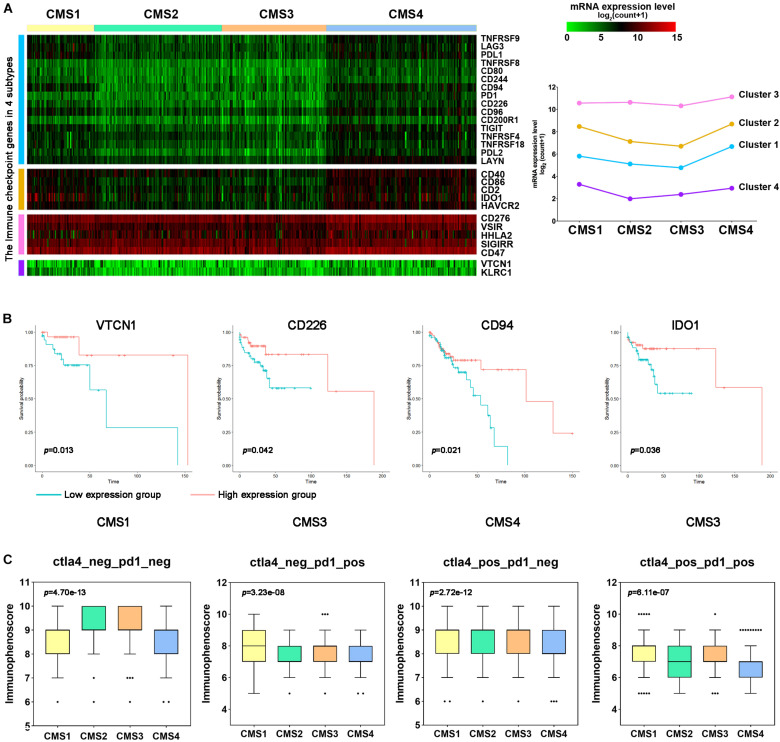
Immune checkpoint gene expression and immune response prediction in each subtype of colon cancer. **(A)** Hierarchical clustering analysis of the mRNA expression levels for immune inhibitory checkpoint-related genes. **(B)** Kaplan–Meier survival curves of immune checkpoint genes (ICGs) in patients with different colon cancer subtypes. Red represents the high expression group, and blue represents the low expression group. **(C)** Comparison of the IPS between patients with each subtype of colon cancer in the CTLA4-negative/positive or PD-1-negative/positive groups. CTLA4 positivity or PD1 positivity represents anti-CTLA4 or anti-PD-1/PD-L1 therapy, respectively.

Immune checkpoint genes play crucial roles in evading self-reactivity and represent a new target to develop cancer treatments (Schoenfeld and Hellmann, 2020; Mauri et al., 2021). First, based on the published literature, we screened 28 ICGs and evaluated the differences in their expression levels among the different subtypes (Figure 8A). We performed hierarchical cluster analysis on the expression levels of these ICGs and evaluated their overall expression levels in the 4 subtypes based on the average expression of 28 genes. The expression levels of most genes in CMS1 and CMS4 were higher. The first cluster of genes showed a higher expression level in CMS4, and the fourth cluster of genes showed a higher expression level in CMS1 (Figure 8A). PDL1, LAG3, KLRC1, CD94, CD244, and IDO1 expression was higher in the CMS1 subtypes (Supplementary Figure 6; *p* < 0.0001). PD1, PDL2, CD2, CD40, CD47, CD80, CD86, CD96, and CD200R1 were higher in the CMS4 subtype than in the other subtypes. The increased expression of ICGs in CMS1 and CMS4 suggests that these two types may respond better to immunotherapy. To further verify this hypothesis, we divided different types of samples into high-expression and low-expression groups according to the expression levels of ICGs and compared the OS between the two groups of samples. The patients with higher expression levels of immune checkpoints had a longer OS. For example, patients with higher levels of VTCN1 expression had a better OS rate in CMS1 (Figure 8B).

Next, we used the immune score to predict the response of the different subtype samples to PD1/PDL1 treatment. In the PD1-positive group, the IPS of CMS1 was higher than that of the other subtypes (Figure 8C). However, the IPS of CMS4 was lower in these groups. The above results indicate that CMS1 patients may have a higher positive response to immunotherapy, such as anti-PD-1/PD-L1 therapy or anti-PD-1/PD-L1 combined with anti-CTLA4 treatment.

## Discussion

The high heterogeneity of colon cancer remains a challenge to treat patients with different types of colon cancer. This study further interpreted the specific molecular characteristics of different molecular subtypes by analyzing the RNA levels, DNA alterations, and immune cell infiltration levels in the four molecular subtypes of colon cancer. First, by examining the RNA levels in the different molecular types, RNAs with specific expression levels in the different subtypes were screened, including mRNAs, lncRNAs, and miRNAs. These subtype-specific RNAs may be biomarkers for patients with different colon cancer subtypes’ clinical diagnosis, treatment, and prognosis. For example, compared with the other subtypes, TDGF1 expression was lower in CMS1. Low TDGF1 expression results in a worse prognosis of patients with the CMS1 subtype (Supplementary Figure 3). Similarly, Sun et al. (2020) reported that TDGF1, an immune-related gene (IRG), is a low-risk prognosis-related gene in colon cancer. Besides, we found that patients with high expression of CLDN8 have a worse prognosis in the CMS1 subtype. Claudin 8 (CLDN8) is a complete membrane protein that constitutes the tight junction of the cell membrane. Cheng et al. (2019) have shown that CLDN8 promotes the proliferation, migration, and invasion of CRC cancer cells by activating MAPK/ERK signaling. Moreover, Gröschl et al. (2013) found that DUSP4 expression was lower in MSS colon cancer samples than MSI-H colon cancer samples by microarray analysis. This result is consistent with our research, which showed low expression of DUSP4 in CMS2 (Supplementary Figure 8). Intelectin-1 (ITLN1) is a novel adipokine that is used as a marker of metabolic disorders. Many studies reported that it might be an effect as an inhibitor in multiple cancers, including gastric cancer, ovarian cancer, and colon cancer. Katsuya reported that colon cancer patients with low ITLN1 expression had a higher grade of metastasis (M grade) than colon cancer patients with retained ITLN1 expression. And patients with retained ITLN1 expression had a better prognosis (Katsuya et al., 2020). This study showed that ITLN1 was significantly higher in CMS3 (a metabolic subtype) than other subtypes (Supplementary Figure 8). Furthermore, an increased expression of ITLN1 showed a better prognosis in colon cancer patients (Supplementary Figure 8). Next, we further analyzed the mutations of subtype-specific RNA, and the expression level of many subtype-specific RNAs was significantly correlated with DNA mutations. For example, the expression level of the specific gene MSH4 in CMS1 is positively correlated with DNA amplification. The expression levels of the IGF2 and IGF2-AS genes in the CMS2 subtype are significantly positively correlated with DNA amplification. These results also indicate that changes in specific genes in different subtypes may help explain the molecular characteristics of different subtypes.

In addition to the impact of subtype-specific genes on the different subtypes, driver gene changes also play essential roles. Many studies have shown that genetic alterations in driver genes contribute to colon cancer development and malignant progression (Nakayama and Oshima, 2019). In 2012, a comprehensive analysis of colon cancer using TCGA found that 16% of colon cancer cases contained hypermutations in microsatellite regions and were classified as having an MSI phenotype (Cancer Genome Atlas Network, 2012a). The other 84% of colon cancer samples were identified as microsatellite stable (MSS) and showed more mutations (Cancer Genome Atlas Network, 2012a). The most frequently mutated genes in colon cancer are APC, KRAS, SMAD4, and TP53 (Huang et al., 2018; Yaeger et al., 2018). Mutations in these genes occur in approximately 10–80% of colon cancers and play crucial roles in CRC metastasis (Cancer Genome Atlas Network, 2012a; Giannakis et al., 2016). Additionally, many other driver genes are frequently mutated in colon cancer, such as ARID1A, SOX9, FAM123B, BCL9L, RBM10, CTCF, and KLF5. In the present study, APC (73%), SMAD4 (66%), ARID1A (47%), and TP53 (46%) were mutated in many colon cancer samples. CMS2, as the most common chromosomal unstable subtype, has significant somatic CNVs. In addition to the higher mutation levels of APC and TP53, our analysis showed that the copy number deletion of TGIF1 in CMS2 was significantly higher than that in the other subtypes. Abnormally high expression of TGIF1 is found in colorectal cancer tissues and promotes the progression of CRC (Shah et al., 2019; Li et al., 2020). And our research showed that ARID1A deletion is more common in CMS4 than in other subtypes. ARID1A is essential for maintaining intestinal stem cells and intestinal homeostasis in mice, and its deletion can induce the occurrence of CRC in mice (Mathur et al., 2017; Hiramatsu et al., 2019). Erfani also proved that ARID1A downregulation might promote CRC metastasis and epithelial cell movement by decreasing EMT-related protein E-cadherin (Erfani et al., 2021). In addition to influencing colon cancer progression, abnormally altered driver genes may help explain the differences among the four subtypes in response to clinical treatment. The mutation frequencies of TP53 were 56, 32, and 59% in the CMS2, CMS3, and CMS4 subtypes, respectively. Based on this result, TP53 mutation indicates a robust carcinogenic effect on most colon cancers (Cancer Genome Atlas Network, 2012b). Therefore, further research into driver gene mutations in colon cancer may have good therapeutic value for targeted therapy.

The proportion of MSI in the CMS1 subtype is significantly higher than that of other subtypes. 15–20% of CMS1 tumors represent MSI characteristics. MSI tumor’s core area and surrounding area are rich in tumor-infiltrating cytotoxic T lymphocytes (CTL) compared with MSS tumor (De Smedt et al., 2015). In this study, numerous immune cells in CMS1, including CD8 + T cells, were significantly higher than other subtypes, consistent with previous research. Several studies have confirmed that immune cell infiltration is especially related to the better prognosis of MSI tumors (Deschoolmeester et al., 2011). Furthermore, the study indicated the presence of CD8 + TILs on the periphery of the tumor was significantly associated with a better prognosis (Park et al., 2016). Therefore, TIL is a critical prognostic component of CMS1. Besides, A high level of M1 macrophage infiltration in patients with the CMS3 subtype indicated a longer survival time. High Treg and NK cell infiltration in the CMS4 subtype indicated a worse prognosis for patients with colon cancer. CAFs were significantly different among the four subtypes of colon cancer and correlated with immune cell infiltration. In the CMS1 immune subtype, CAFs were negatively correlated with T cell infiltration. We assessed the correlation between immune cells and the TMB. M1 and M2 macrophages showed a positive correlation with the TMB in the CMS3 subtype (Supplementary Figure 5). These results indicated that immune cells were correlated with the survival of patients with different subtypes of colon cancer.

Since ctLA-4 inhibitors were approved for melanoma in 2011, PD1/PD-L1 inhibitors have now been used to treat more than 20 different cancers, including colon cancer. In the present study, we analyzed 29 ICGs and found that most were expressed at higher levels in CMS4 than in the other subtypes, such as PD1, PD-L1, PD-L2, CD2, and CD40. Most ICGs showed low mutation levels in colon cancer cases, except CD2, VTCN1, and LAYN (Supplementary Figure 7). This result suggests the stability of immune therapy targeting ICGs. The CMS1 subtype responds well to anti-PD-1/anti-CTLA4 combination treatment but not to anti-CTLA4 monotherapy. However, the other subtypes did not respond to either anti-CTLA4 or anti-PD-1 therapy. These features suggest that the CMS1 subtype is susceptible to immunotherapy. Significant immune responses were observed in patients with different colon cancer subtypes, a finding that may be related to the complex cellular components in tumor tissues. In the future, the effect of tumor immunotherapy may be improved by changing the ratio of immune cells. The interplay mechanisms of tumor cells and immune cells in different subtypes are essential to predict the therapeutic response and prognosis. They may also contribute to developing an individualized treatment plan for patients with different subtypes of colon cancer.

In summary, we investigated the specific molecular characteristics of the four subtypes of colon cancer from multiple perspectives. The findings may provide a theoretical basis to identify patients more likely to benefit from immunotherapy and offer potential biomarkers for future immunotherapy.

## Data Availability Statement

The datasets presented in this study can be found in online repositories. The names of the repository/repositories and accession number(s) can be found in the article/Supplementary Material.

## Author Contributions

JYW and FH designed and supervised this study. FH drafted the manuscript. JYW and SW illustrated the figures for the manuscript and analyzed the data. LZ and MZ searched the database. BC, HY, and JRW reviewed and edited the manuscript. All authors approved the final manuscript.

## Conflict of Interest

The authors declare that the research was conducted in the absence of any commercial or financial relationships that could be construed as a potential conflict of interest.

## Publisher’s Note

All claims expressed in this article are solely those of the authors and do not necessarily represent those of their affiliated organizations, or those of the publisher, the editors and the reviewers. Any product that may be evaluated in this article, or claim that may be made by its manufacturer, is not guaranteed or endorsed by the publisher.

## Footnotes

1
https://xenabrowser.net/datapages/

2
http://metascape.org/gp/index.html

3
http://ngs.ym.edu.tw/driverdb

4
http://www.pathwaymapper.org/

5
https://www.cbioportal.org/

6
https://github.com/icbi-lab/Immunophenogram
