# Supplementary figures and images for: Comprehensive Analysis of Subtype-Specific Molecular Characteristics of Colon Cancer: Specific Genes, Driver Genes, Signaling Pathways, and Immunotherapy Responses
Source: Front Cell Dev Biol. 2021 Nov 29;9:758776. doi: 10.3389/fcell.2021.758776 (PMC8667669; doi:10.3389/fcell.2021.758776)

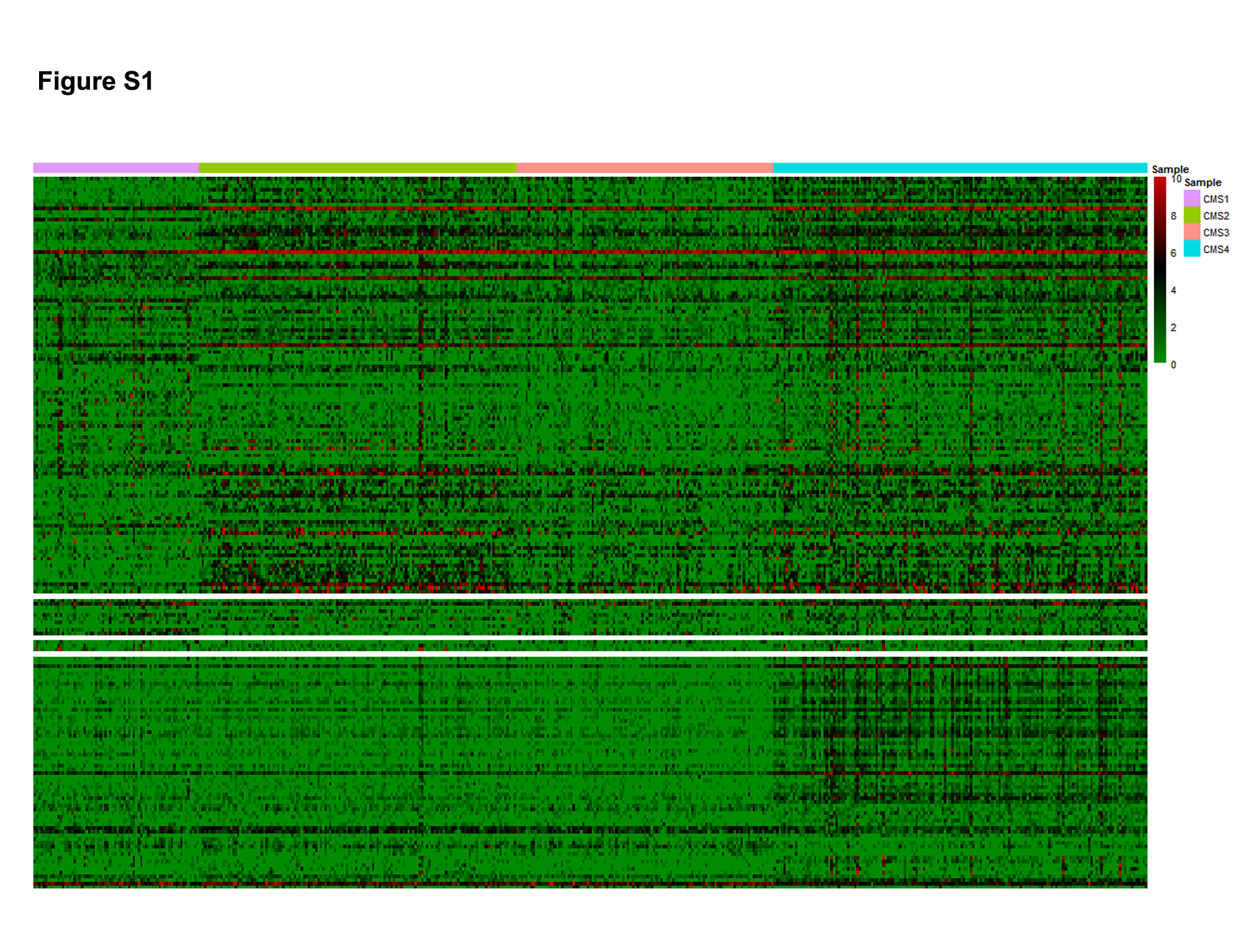

Supplement: Supplementary file 1 [file Image_1.TIFF]

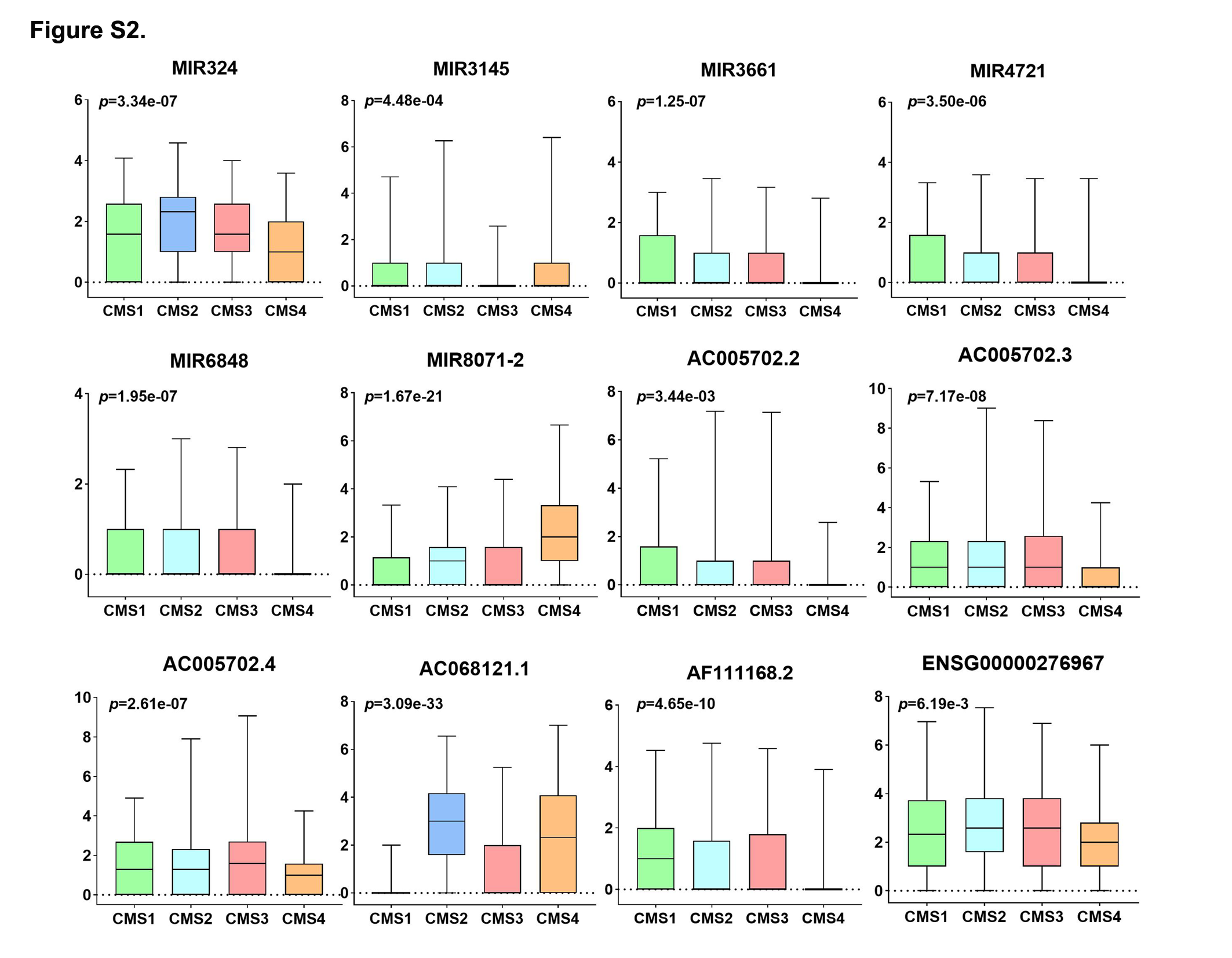

Supplement: Supplementary file 2 [file Image_2.TIF]

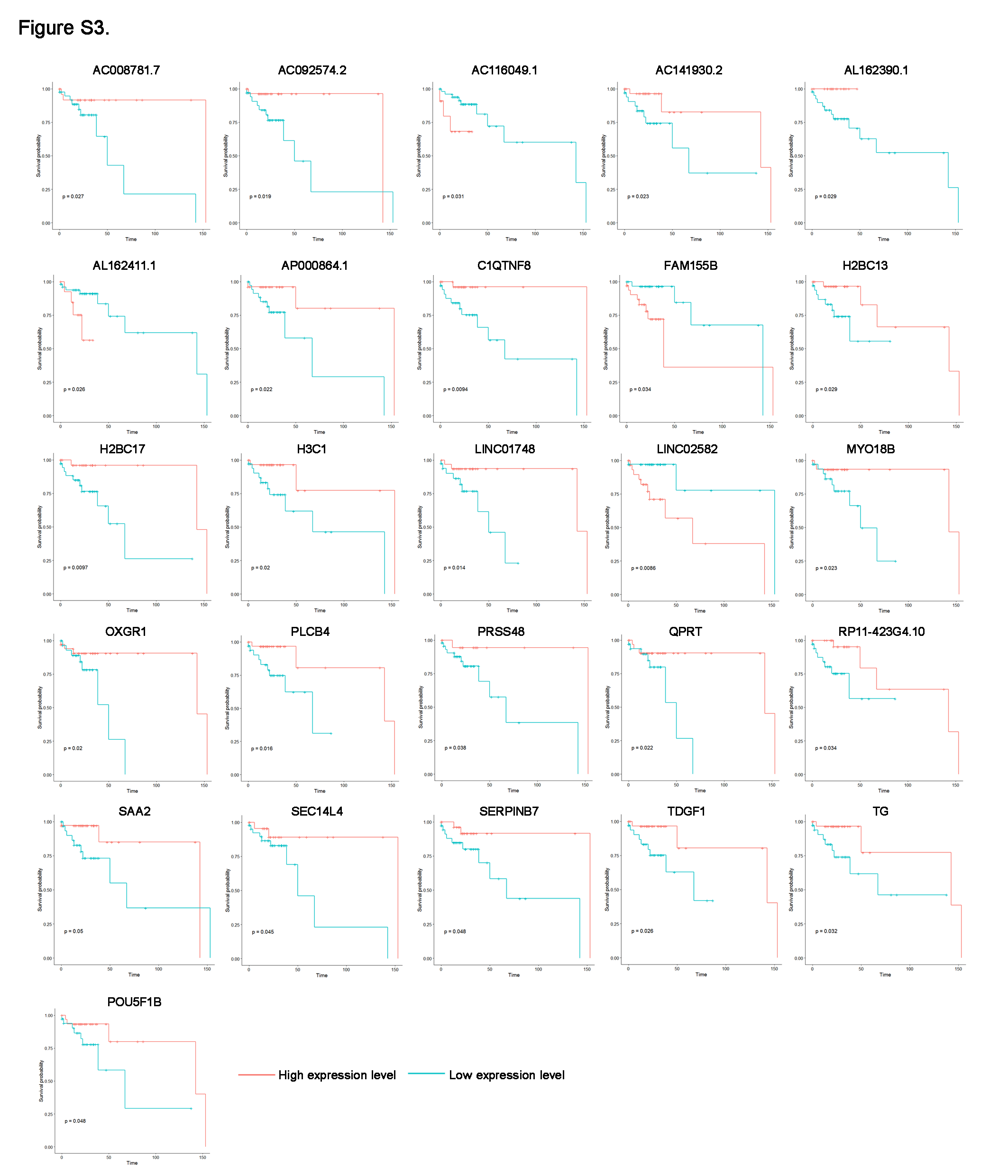

Supplement: Supplementary file 3 [file Image_3.TIF]

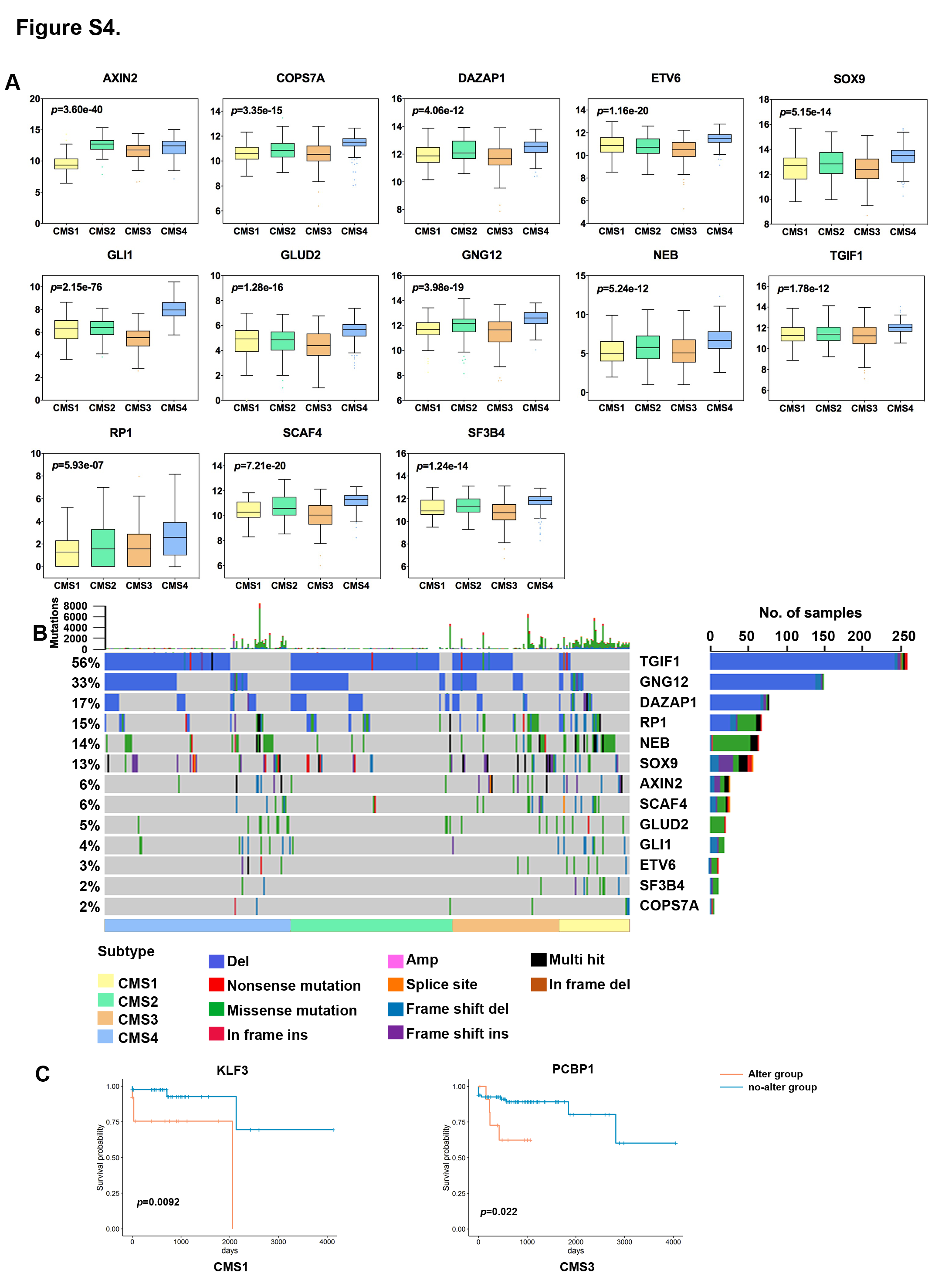

Supplement: Supplementary file 4 [file Image_4.TIF]

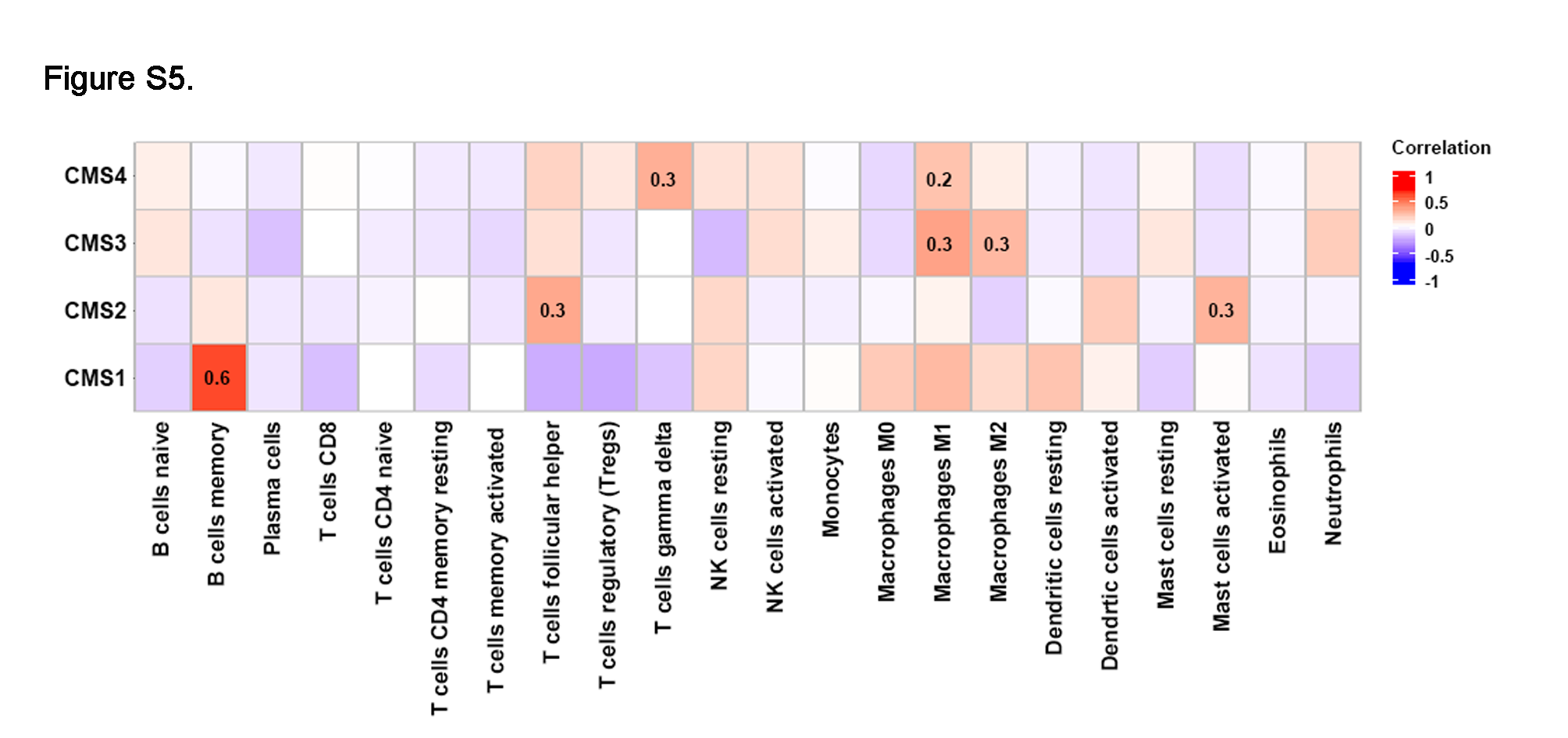

Supplement: Supplementary file 5 [file Image_5.TIF]

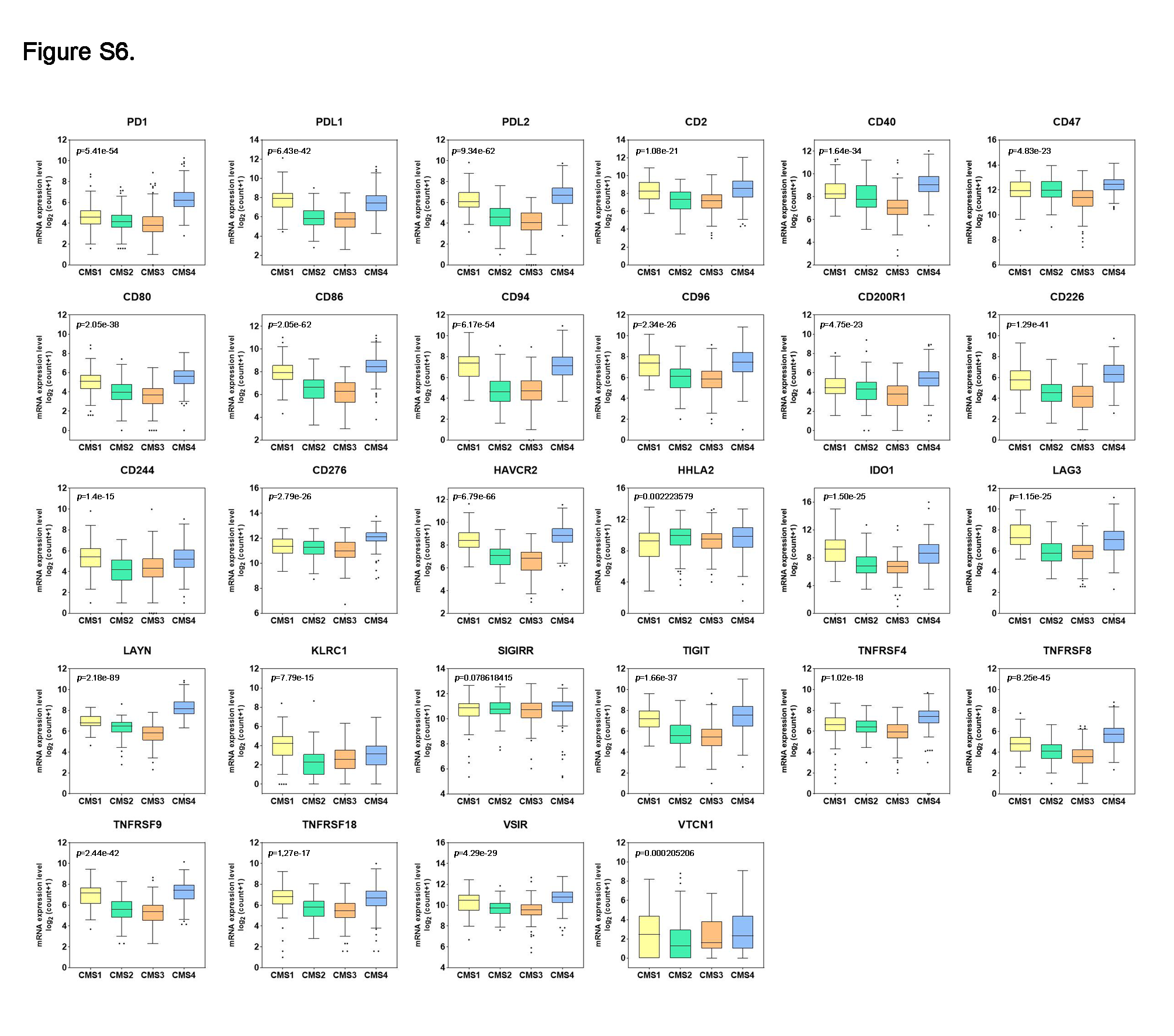

Supplement: Supplementary file 6 [file Image_6.TIF]

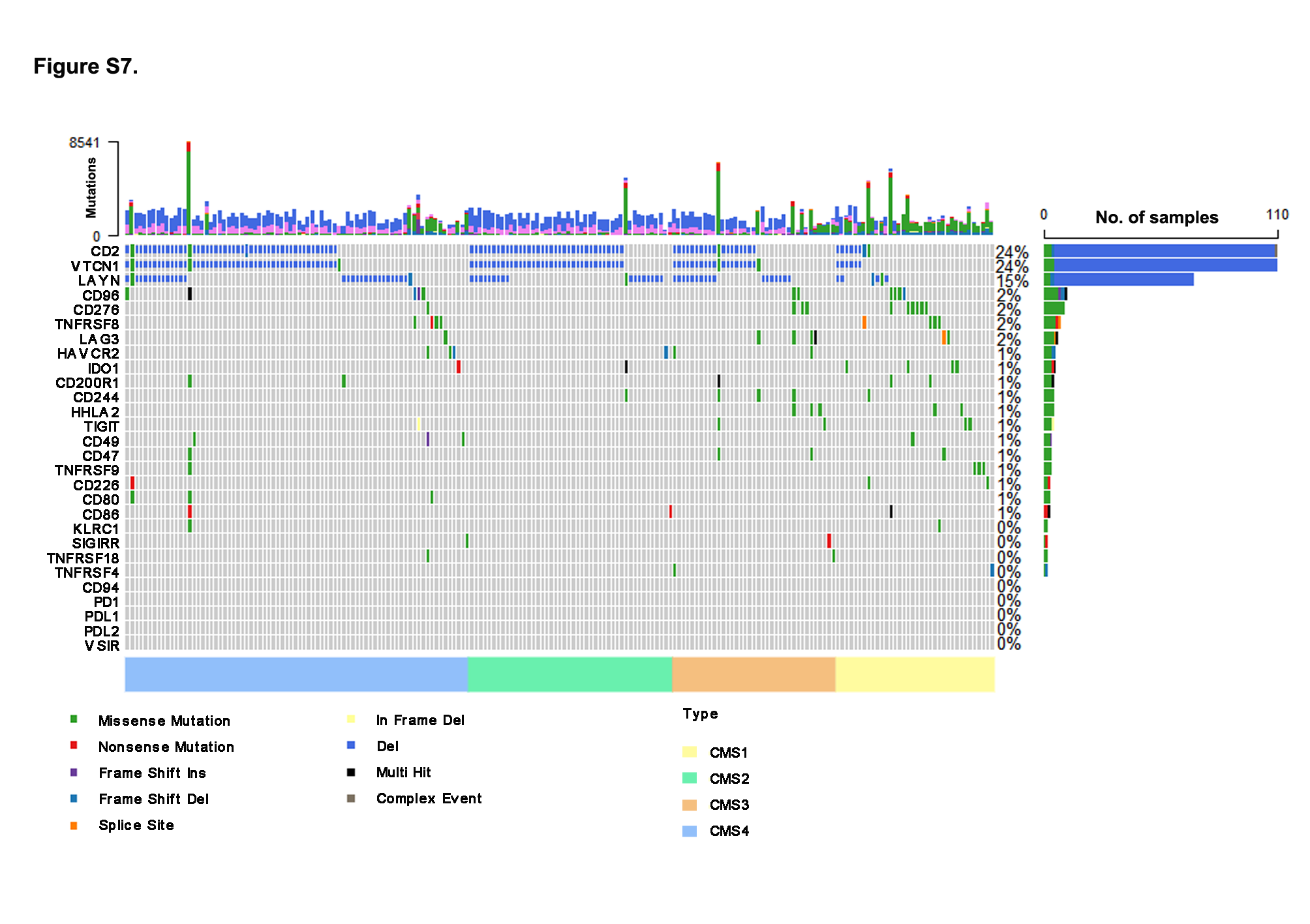

Supplement: Supplementary file 7 [file Image_7.TIF]

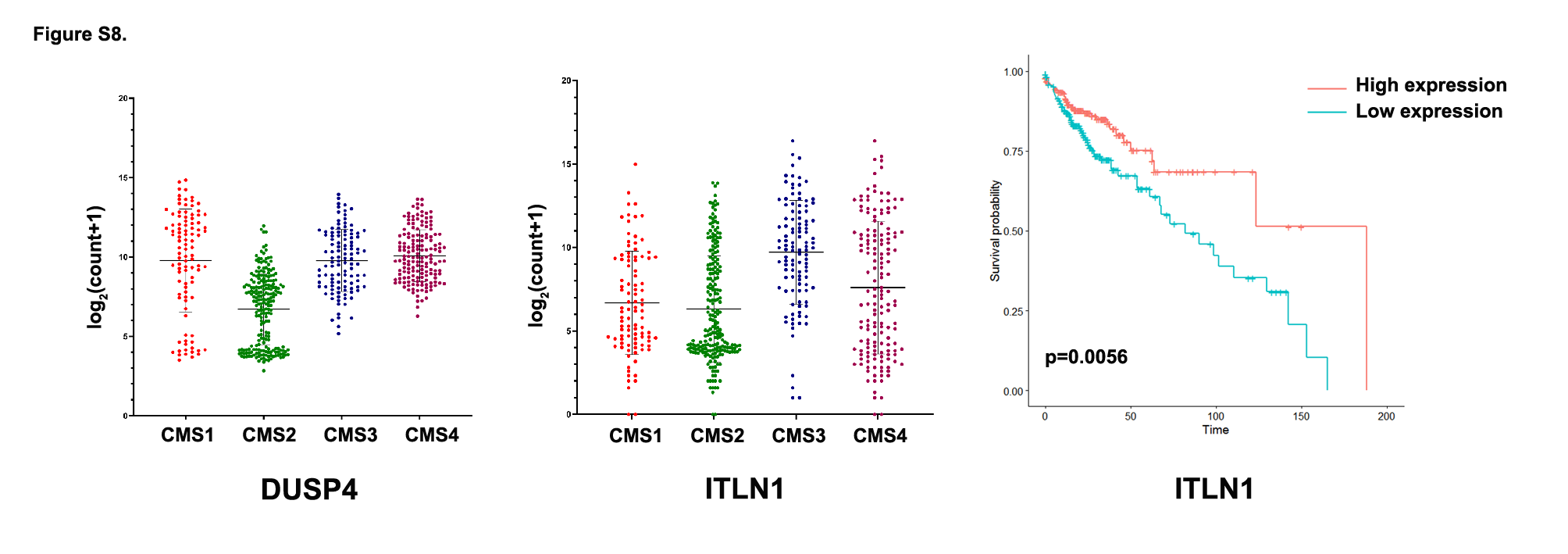

Supplement: Supplementary file 8 [file Image_8.TIF]
