# Supplementary material for: Comprehensive Analysis of Subtype-Specific Molecular Characteristics of Colon Cancer: Specific Genes, Driver Genes, Signaling Pathways, and Immunotherapy Responses
Source: Front Cell Dev Biol. 2021 Nov 29;9:758776. doi: 10.3389/fcell.2021.758776 (PMC8667669; doi:10.3389/fcell.2021.758776)
Supplement: Supplementary file 11 [file Table_3.DOC]

Supplement Figure Legends

Figure S1. Comparison of specific lncRNA expression levels among the colon cancer

subtypes.

Figure S2. Comparison of specific miRNA expression levels among the colon cancer

subtypes.

Figure S3. Kaplan-Meier survival curves were generated for subtype-specific RNAs by comparing groups with high (red line) and low (blue line) gene expression. *p*< 0.05 according to the log-rank test.

Figure S4. Analysis of the driver genes in different colon cancer subtypes. A. Expression levels of the driver genes in each subtype. B. The waterfall chart shows the frequency of mutations in the driver genes in each subtype. C. Kaplan-Meier survival curve of the driver genes with gene mutations. Red indicates the gene alteration group, and blue indicates the nonaltered group.

Figure S5. Correlation analysis between the TMB and levels of twenty-two infiltrating immune cells in different subtypes. Red indicates a positive correlation, and blue indicates a negative correlation. The number represents the degree of correlation, and *p*<0.05.

Figure S6. Comparison of immune checkpoint gene expression levels between each subtype of colon cancer.

Figure S7. Waterfall chart showing the immune checkpoint gene mutations in different subtypes of colon cancer.

Supplement Table 1. The speical RNA in four subtype of colon cancer.

Supplement Table 2. The expression level of 30 driver genes in different subtypes.
